# Influence of Pentaerythritol Tetraacrylate Crosslinker on Polycarboxylate Superplasticizer Performance in Cementitious System

**DOI:** 10.3390/ma15041524

**Published:** 2022-02-18

**Authors:** Yu Gao, Hongwei Zhao, Guang Chen, Qi Peng, Yingying Liu, Fei Song, Qingquan Liu

**Affiliations:** 1 School of Material Science and Engineering, Hunan University of Science and Technology, Xiangtan 411201, China; g18707328127@163.com (Y.G.); cghnust@163.com (G.C.); pengqi408@126.com (Q.P.); h1486021522@163.com (Y.L.); songfei@hnust.edu.cn (F.S.); 2 Hunan Provincial Key Laboratory of Advanced Materials for New Energy Storage and Conversion, Hunan University of Science and Technology, Xiangtan 411201, China

**Keywords:** polycarboxylic, crosslinked, superplasticizer

## Abstract

In this work, a crosslinked polycarboxylate superplasticizer (crosslinked-PC) was synthesized via the free radical polymerization reaction. Pentaerythritol tetraacrylate (PETA) was used as the crosslinked agent. A comparative comb-like polycarboxylate superplasticizer (comb-like-PC) was prepared under the same reaction conditions. The dispersion retention capacity, dispersion capability, hydration characteristics of the cement paste and setting time were investigated in detail. At the dosage of 0.6% bwoc, the fluidity of the cement/crosslinked-PC paste was about 340 mm, which was 40~50 mm larger than the cement/comb-like-PC paste. The dispersion retention capacity of the cement/crosslinked-PC paste was observed to be much superior due to higher adsorbed amounts on the cement particles. Moreover, the cement/crosslinked-PC paste exhibited the initial and final setting durations of 196 and 356 min, respectively, which indicated an enhancement of 18 and 68 min compared to the cement/comb-like paste. The crosslinked copolymers exhibit a stronger retardation effect than the comb-like copolymers due to their enhanced adsorbed amounts and stronger steric hindrance effect. This is further illustrated by the characterization of the hydration process and hydration products. It can be concluded that it is feasible to improve the dispersive capacity and the dispersion retention capacity of PC by changing the molecule structure from comb-like to slightly crosslinked.

## 1. Introduction

The polycarboxylic superplasticizers (PCs) have been widely applied in the field of commercial concrete. As China has emerged as the world’s largest construction market, it has also boosted the application of superplasticizers. PCs have become the essential components of concrete owing to its advantages such as its low dosage, notable dispersive and dispersion retention capacity, environmental friendliness and designability of molecular structure [1,2,3,4,5,6]. In general, the PC molecules show a comb-like structure, which consists of a linear backbone with polyethylene glycol side chains and carboxylate [7,8,9,10]. The PC molecules could adsorb on the surface of cement particles, thereby hindering the aggregation of cement particles due to the steric hindrance of the long polyethylene glycol chains [11,12,13,14]. With the increasing demand for the performance of commercial concrete, it is very important to develop PCs with novel structure to satisfy the practical application requirements. Thus, the exploration of a diversified design of PC structure is an important scientific issue that is attracting significant research attention.

With the advancements in molecular structure diversification, the molecular structure design for PCs has become a focus of research for attaining a superior performance. A number of research studies have reported the synthesis of novel PCs with the crosslinked molecular structures, with much improved performance. Etsuo et al. controlled slump loss by introducing the diethylene glycol diacrylate as a crosslinking agent in PC copolymers [15]. Cai et al. found that a crosslinked polycarboxylate superplasticizer could effectively improve the fluidity of mortar and maintain its fluidity as well [16]. Liu et al. reported the synthesis of the CLPCs. The developed CLPC performed an obvious slow-release function and demonstrated a higher paste flow after 150 min than the initial material [17]. Liu et al. designed and prepared an “octopus-like” PC with a star-shaped structure, in which each arm consisted of polycarboxylate and polyethylene glycol [18]. Thus, PCs can theoretically achieve strong steric hindrance, which significantly improves their effectiveness and performance. Lin et al. explored the influence of two crosslinked polycarboxylate superplasticizers. It was observed that the superplasticizer with the linear crosslinking agent imparted the cement paste samples higher water reduction rate, superior dispersion effect and greater retardation effect than the uncrosslinked superplasticizer and the superplasticizer with a macrocyclic crosslinking agent [19].

In this work, a crosslinked polycarboxylate superplasticizer (crosslinked-PC) was synthesized. Pentaerythritol tetraacrylate (PETA) was used as the crosslinked agent. The dispersion maintaining ability, dispersion capability, hydration characteristics of the cement paste and setting time were investigated in detail. Additionally, the adsorption behavior of crosslinked-PC was investigated by measuring the adsorption amount, and the working mechanism of the crosslinked-PC in the cementitious system was further summarized.

## 2. Materials and Methods

**Figure 1:**
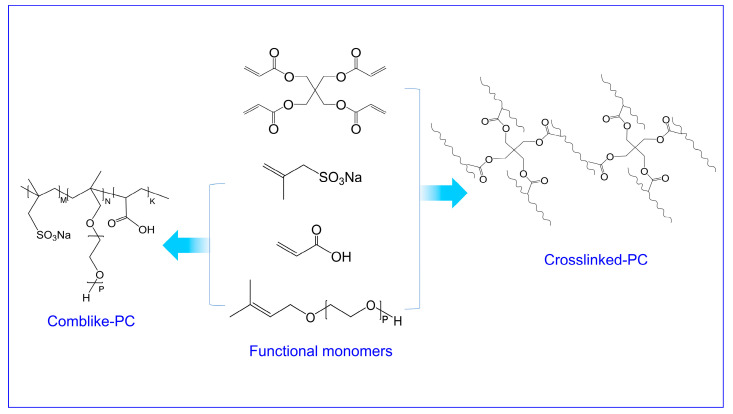
Synthetic routes for the preparation of the comb-like-PC and crosslinked-PC.

Materials Pentaerythritol tetraacrylate (PETA), acrylic acid (AA), potassium peroxodisulfate (KPS), sodium methylallyl sulphonate (SAMS) and thioglycolic acid (TGA) were provided by Aladdin Industrial Corporation (Shanghai, China). The methyl allyl polyethylene glycol (HPEG) (M_w_ = 2200 g/mol) was provided by Taijie Chemical Co., Ltd. (Shanghai, China). The cement (P.I 42.5) was supplied by China United Cement Corporation (Beijing, China). AA, HPEG, SAMS and TGA were added to a flask and dissolved in 40 mL deionized water through the continuous agitation reactor. The crosslinker PETA and initiator KPS were mixed completely in deionized water (30 mL) and then added to the flask by peristaltic pump at a rate of 0.5 mL/min. The polymerization was carried out at 75 °C for four hours. The prepared sample was denoted as crosslinked-PC. A comparative sample was performed in the absence of PETA under the same reaction conditions and which was denoted as comb-like-PC. The aqueous products were neutralized to pH = 7 by NaOH solution. The synthetic routes of the crosslinked-PC and comb-like-PC are shown in Figure 1 and the monomer combination of PCs are shown in Table 1.

**Methods Fourier Transform Infrared (FTIR) Measurement.** FTIR spectra were measured with an FTIR spectrometer (Nicolet 570, Madison, WI, USA). The spectra were recorded with a spectral range of 400~4000 cm^−1^. All samples were dried at 60 °C for 12 h in a vacuum oven before FTIR measurements.

**Gel Permeation Chromatography (GPC) Measurement.** The molecular weights were measured with Waters 1525/2414 instrument (Waters, Milford, MA, USA). PEG was used as the calibration standard, the measurement was performed at 25 °C.

**^1^H-NMR Measurement.** The ^1^H-NMR results were measured with a 400 MHz DRX-400 spectrometer (Bruker, Karlsruhe, Germany). Samples for measurement were dissolved in deuteroxide. The chemical shift values were expressed in δ values (ppm) relative to tetramethylsilane (TMS) as an internal standard.

**Cement Paste Fluidity Measurement.** Cement paste fluidity test was measured depending on the standardization of GB/T 8077-2012 [20]. The dosages of PC were added in the range of 0.1~0.7% (solid amount) by weight of cement (bwoc) with a water/cement (w/c) ratio of 0.35. The time dependent fluidity loss was carried out depending on the standardization of GB/T 50080-2002 [21].

**Total organic carbon (TOC) Measurement.** The adsorbed amounts of the PCs on cement particles were measured with a TOC analyzer (Multi N/C3100, analytikjene AG, Jena, Germany). In total, 10 g blank cement was mixed with superplasticizer solution (0.1 to 0.7% bwoc, 40 g), respectively. Then, the cement paste was centrifuged at 5000 rpm for 10 min. The supernatant solution was diluted with deionized water for TOC measurement. Each sample was tested thrice, the average value was calculated.

**Setting Time.** The initial and final setting time of cement pastes were determined by a Vicat analyzer depending on the standard GB/T 1346-2011 [22]. The initial setting was recorded as the Vicat needle was 4 ± 1 mm from the floor, the final setting was recorded as the Vicat needle penetrated 0.5 mm into the cement paste. Each sample was tested thrice, and the average value was calculated.

**Isothermal Calorimetry Measurement.** The hydration heat was measured with an isothermal conduction calorimeter(TAMair, Thermometric, Jarfalla, Sweden) for 7 days. In total, 10.0 g cement was filled into 20 mL glass ampoules, mixed with the PC solution (w/c = 0.4).

**Rheological Measurements.** Rheometric measurement of cement pastes were performed by a DHR Rheometer (TA Instruments, Newcastle, DE, USA) using a vane fixture to assess changes in paste viscosity.

**X-ray Diffraction (XRD) Measurement.** X-ray diffraction was measured with a D8 ADVANCE power diffractometer (Bruker, Karlsruhe, Germany), the diffraction angle (2θ) was from 2 to 50°.

**Compressive Strength Measurement.** Compressive strength measurements were determined by a TYE-2000B machine(Luda Construction Instrument Co., LTD, Tianjin, China). The cement mixture (w/c = 0.35) was fed into a 7 cm × 7 cm × 7 cm mold. The cement block was hydrated for 7 days.

**Scanning electron microscope (SEM) Measurement.** The morphology of the hydration products was measured with a scanning electron microscope (JSM-5900, JEOL Co. Ltd., Tokyo, Japan). Ethanol was utilized to discontinue the hydration process.

## 3. Results and Discussion

### 3.1. Structure of the Comb-like-PC and Crosslinked-PC

**Figure 2:**
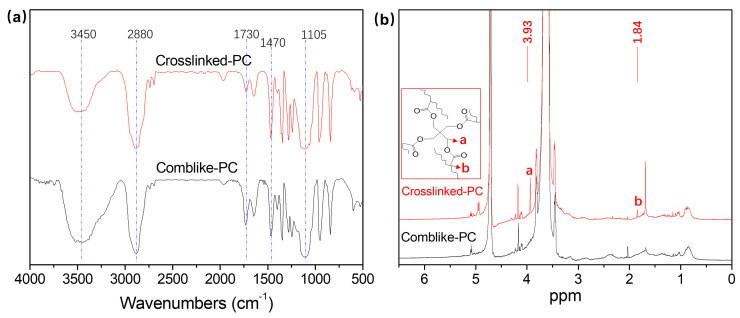
FTIR (**a**) and ^1^H−NMR (**b**) spectra comb-like-PC and crosslinked-PC.

Figure 2a shows the FTIR spectra of the comb-like-PC and crosslinked-PC. For both comb-like-PC and crosslinked-PC, the absorption signals at 1105 and 2880 cm^−1^ attributed to the C-O-C vibration and stretching vibration of polyethylene glycol, respectively [18]. Furthermore, the absorption signal at 1730 cm^−1^ corresponded to the binding of -C=O for carboxylic acid. The peak at 3450 cm^−1^ was attributed to the stretching vibration of the -OH and the signals at 1470 cm^−1^ were due to C-H bending vibrations, respectively [23]. These results demonstrated the existence of the carboxyl and polyethylene glycol in both comb-like-PC and crosslinked-PC structure.

Figure 2b shows the ^1^H-NMR spectra of the comb-like-PC and crosslinked-PC. For all samples, the peaks between 3.4~3.8 and 4.5~5.0 ppm corresponded to the H atoms of polyethylene glycol [18]. Crosslinked-PC shows a characteristic peak (a) at 3.93 ppm corresponding to the H atoms of -CH_2_- in crosslinker PETA but no peak at this position for comb-like-PC. Furthermore, the characteristic peak (b) at 1.84 ppm in crosslinked-PC also does not exist in comb-like-PC, which belonged to the H atoms of double bonds in PETAs’ structure. The aforementioned characteristic peaks of the H atoms in PETA demonstrated that the crosslinker was introduced to the crosslinked-PC successfully.

**Figure 3:**
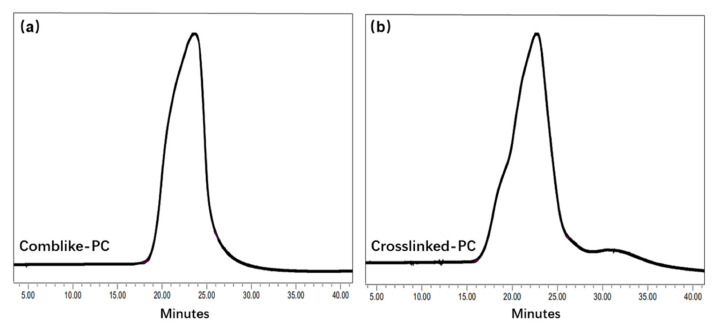
GPC chromatograms of comb-like-PC (**a**) and crosslinked-PC (**b**).

The molecular weight of comb-like-PC and crosslinked-PC were analyzed by GPC measurement. The respective GPC curves are displayed in Figure 3, and the molecular weight and polydispersity (PD) values are calculated in Table 2. The PD values of the comb-like-PC and crosslinked-PC were determined as 2.55 and 2.82, respectively. Furthermore, compared to the comb-like-PC, the crosslinked-PC showed a higher molecule weight. This is mainly due to the formation of a slightly crosslinking network, the crosslinker PETA plays a role in connecting comb molecular chains. Thus, FTIR, ^1^H-NMR and GPC results demonstrated that the PCs with different structures were prepared successfully.

### 3.2. Fluidity and Time-Dependent Fluidity Loss

**Figure 4:**
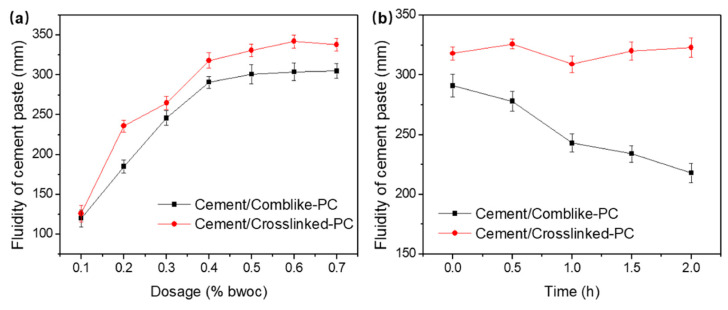
Fluidity (**a**) and time-dependent fluidity loss (**b**) of the cement/comb-like-PC and cement/crosslinked-PC pastes.

Figure 4 shows the fluidity of the cement/PC paste varying with the dosage. Compared to the cement/comb-like-PC paste, the cement/crosslinked-PC exhibited a higher fluidity in the dosage range of 0.1~0.7% bwoc. The saturated dosages of the comb-like-PC and crosslinked-PC were observed to be about 0.6% bwoc. At the dosage of 0.6% bwoc, the fluidity of the cement/crosslinked-PC paste was about 340 mm, which was 40~50 mm larger than the cement/comb-like-PC paste. The result indicated that the crosslinked-PC exhibited a higher dispersive capacity than the comb-like-PC. The dispersive capacity of the PCs is mainly attributed to the steric hindrance of polyethylene glycol side chains. The polyethylene glycol chains can form stable hydrophilic adsorbed layers on the surface of cement particles. As the crosslinked-PC consisted of the crosslinker PETA and comb-like molecule chains, thus, it could be inferred that the crosslinked-PC showed a superior steric hindrance effect than the comb-like-PC.

**Figure 5:**
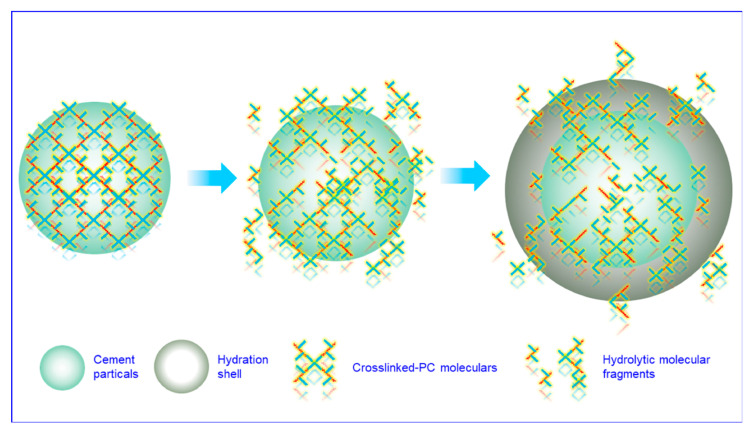
Schematic representation of the dispersion and adsorption behaviors of crosslinked-PC on cement particles.

To evaluate the performance of the superplasticizers, the dispersion retention capacity is another important factor. Dispersion retention capacity is defined as the decrease in fluidity over time. Thus, the change in fluidity was recorded over 2 h. As shown in Figure 4b, the dispersion retention capacity of the cement/crosslinked-PC paste was observed to be much superior than the comb-like-PC. The fluidity of the cement/crosslinked-PC paste was substantially unchanged, while the cement/comb-like-PC paste was noted to drop about 70 mm after 2 h. For the crosslinked-PC, the steric hindrance of the crosslinked-PC played an important role in the dispersion retention capacity of the cement pastes. As the comb-like molecule chains grafted on the crosslinker PETA, the crosslinked emanative structure exhibited a much stronger steric hindrance compared to the linear comb-like molecular chains. Thus, the steric hindrance effect provided by the crosslinked-PC maintained the dispersibility of the cementitious system, which was in accordance with the paste fluidity results. Moreover, due to the existence of Ca(OH)_2_, cement suspension shows strong alkalinity. Thus, it can be inferred that at the initial stage of hydration, the ester groups from crosslinker PETA could be hydrolyzed into carboxylate radical in the cement suspension. As shown in Figure 5, while the crosslinker was broken, the crosslinked structure can be hydrolyzed into comb-like molecular fragments. Thus, a secondary dispersion of the hydrolytic fragments was carried out in the suspension, which improved the dispersion retention capacity of the cement pastes [24]. However, the exact mechanism of the dispersion of crosslinked-PC in the cementitious system requires further investigation.

### 3.3. Adsorption on Cement Particles

**Figure 6:**
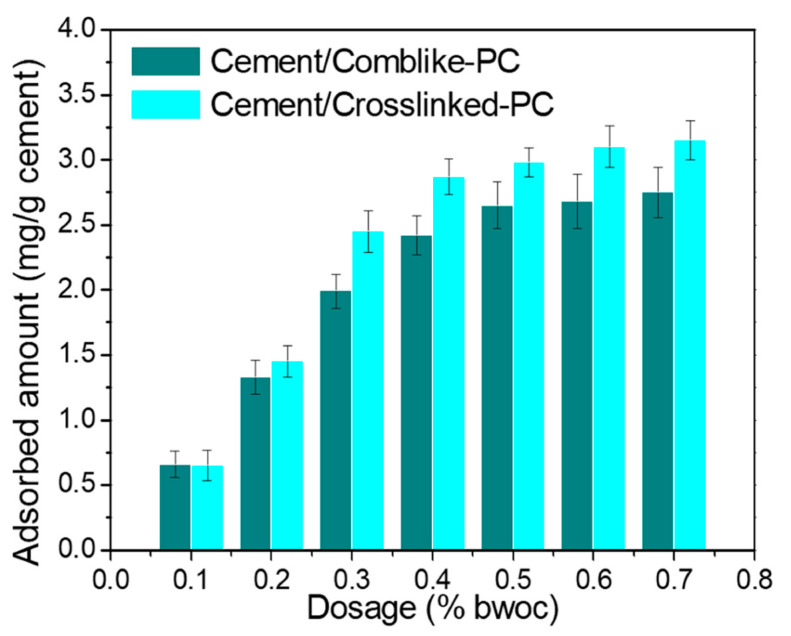
Absorbed amounts comb-like-PC and crosslinked-PC in cement paste at various dosage (% bwoc).

Figure 6 exhibits the adsorbed amounts of the PCs in the cement pastes at different dosages. For the comb-like-PC, the extent of adsorption is observed to increase with the PC dosage, followed by reaching a plateau at a dosage of 0.5% bwoc. Compared with the comb-like-PC, the crosslinked-PC exhibited higher adsorbed amounts. It has been reported that the dispersive capacity of cement paste is mainly due to the adsorption on the surface of the cement particles, and the preferred adsorbate is noted to be the hydrating ettringite (AFt) [15,25,26]. The dispersive capacity of PC is well related to the adsorbed amounts and can be interpreted on the basis of the surface coverage [27]. The crosslinked structure shows a stronger steric hindrance than the comb-like structure, which leads to a high content of the absorbable polar groups in copolymers; thus, a high probability of adsorption on the surface of cement particles occurred [28].

### 3.4. Setting Duration of the Cement Pastes

**Figure 7:**
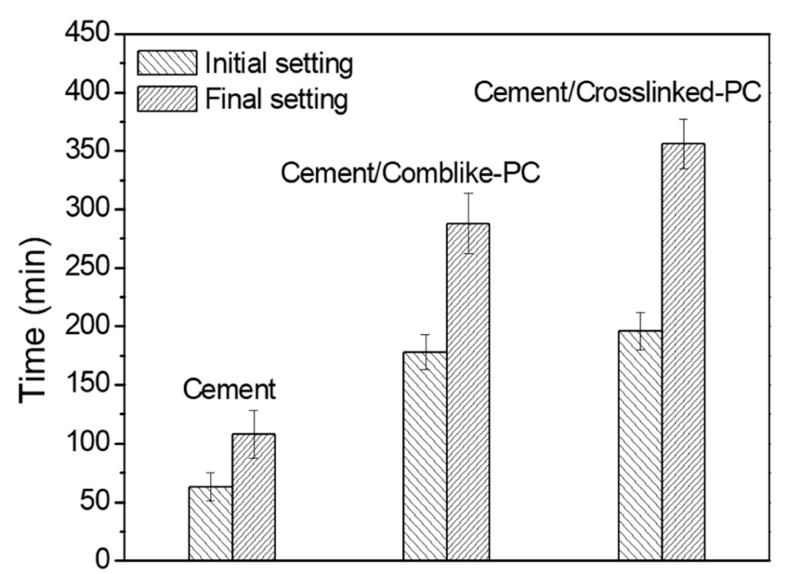
Setting times of cement pastes in the absence or presence of PCs (0.5% bwoc).

The setting time is directly related to the cement hydration process [29]. As soon as the water was added to the cement, the complex hydration reactions started and the cement pastes began to coagulate and harden. As shown in Figure 7, the initial and final setting times of the blank cement paste were 63 and 105 min, respectively. The setting time of the cement/comb-like-PC paste was significantly extended. Furthermore, the cement/crosslinked-PC paste exhibited the initial and final setting durations of 196 and 356 min, respectively, which indicated an enhancement of 18 and 68 min as compared to the comb-like-PC. The observed phenomenon could be attributed to enhanced adsorption amounts of the crosslinked-PC on the surface of the cement particles. A high extent of calcium chelated complexes as well as a remarkable inhibition of the hydration process were observed for the crosslinked-PC, resulting in the retardation of the hydration process [30].

### 3.5. Hydration Heat Flow of Cement

**Figure 8:**
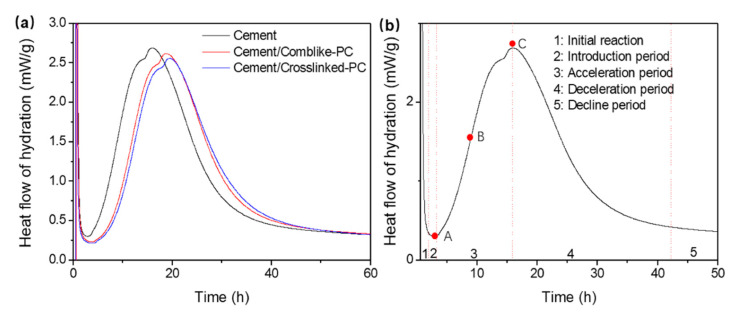
The hydration exothermic heat flow curves of cement/PC samples (0.5% bwoc) (**a**) and blank cement samples (**b**).

The exothermic heat flow curves of the hydration process are shown in Figure 8a. As observed, the addition of the PCs had a retardation effect on the hydration process of the cement pastes. Moreover, compared to the comb-like-PC, the crosslinked-PC exhibited a more noticeable retardation effect, which contributed to the dispersion retention capacity of the cement paste [31].

The parameters of the hydration kinetics were obtained from the exothermic heat flow curves in Figure 8a and the parameters are listed in Table 3. Figure 8b shows the heat flow curve of the blank cement paste. In general, the hydration process can be divided into five stages [26,32], the starting point of the acceleration period was denoted as *t*_A_, the end point of the acceleration period was denoted as *t*_C_, and the point of the maximum acceleration rate was denoted as *t*_B_. Meanwhile, the heat generation rate during the induction period was denoted as (d_Q_/d_t_)_A_, and the maximum hydration rate in the acceleration period was denoted as (d_Q_/d_t_)_C_, respectively. *k*_A–B_ corresponds to the secant slope on the exothermic heat flow curve between A and B, which means the acceleration rate in the acceleration period. As noted in Table 3, the comb-like-PC and crosslinked-PC evidently delayed the induction period, implying the ion diffusion was decelerated due to the adsorption of the comb-like-PC and crosslinked-PC on the surface of cement particles. Moreover, the crosslinked-PC shows a more evident retardation of the cement hydration process than the comb-like-PC, which was consistent with the results that showed the adsorption amounts of the crosslinked-PC were higher than those of the comb-like-PC. As compared to the blank cement paste, k_A–B_ and (d_Q_/d_t_)_C_ decreased on the addition of the comb-like-PC and crosslinked-PC. Moreover, the comb-like-PC led to a weaker retardation effect than the crosslinked-PC, thus, suggesting that crosslinked-PC had a strong impact on delaying the generation of the hydrates during the acceleration period. Thus, the crosslinked copolymers exhibit a stronger retardation effect than the comb-like copolymers due to their enhanced adsorption amounts and strong steric hindrance effect.

### 3.6. Rheology Behavior of Cement Pastes

**Figure 9:**
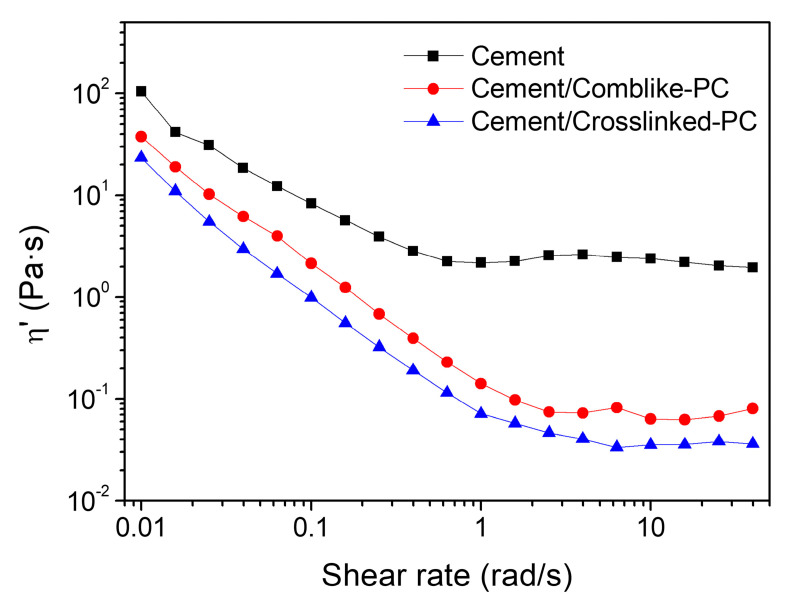
The viscosity η’ versus shear rate variations in the cement pastes at the PC dosage of 0.5% bwoc.

The addition of PC has an influence on the rheological response of cement paste [33]. Figure 9 shows the viscosity *η*’ of cement pastes (w/c = 0.35) with the PC dosage of 0.5% bwoc. A shear thinning behavior was observed for all samples from a shear rate of 0.01~50 rad/s. With a dosage of 0.5% bwoc, the cement paste containing cement/comb-like-PC and cement/crosslinked-PC had a viscosity at 0.01 rad/s of 37.6 and 23.5 Pa s, respectively. Cement paste had the highest viscosity at 0.01 rad/s at 105.2 Pa s, which is about 3 and 4 times than that of cement/comb-like-PC and cement/crosslinked-PC paste, respectively. This suggests that the crosslinked-PC led to a more significant sharp reduction in yield stress than comb-like-PC, which is consistent with the results of fluidity. With the increase in shear rate, a clear shear thinning behavior for the pastes occurred. Thus, the addition of crosslinked-PC sharply decreased the aggregation of cement pastes and resulted in the transition from the solid-like to liquid-like viscoelastic behaviors.

### 3.7. XRD and Compressive Strength Analysis of Hydration Products

**Figure 10:**
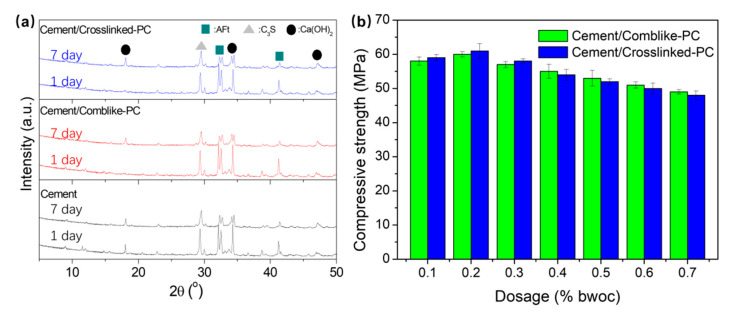
WAXD patterns (**a**) and the compressive strengths (**b**) of the hydration products.

The main components of cement are tetracalcium aluminoferrite (C_4_AF), dicalcium silicate (C_2_S), tricalcium aluminate (C_3_A) and tricalcium silicate (C_3_S). The hydration rates of C_3_S, C_3_A and C_4_AF are faster than C_2_S [34]. The solid phase dispersed, disintegrated and then suspended in the liquid phase. In the early stage of the hydration process, the products contained needle-like AFt phase, square Ca(OH)_2_ crystals and amorphous C-S-H gel. The XRD patterns of cement hydration products are shown in Figure 10a. The diffraction peaks at 2θ = 18.0, 34.0 and 46.9^o^ occurred due to the formation of the Ca(OH)_2_ crystals [18]. For the samples hydrated for 1 day, the cement/comb-like-PC exhibited a weak signal at 2θ = 18.0^o^, while the signal in cement/crosslinked-PC could hardly be observed, which indicated that the addition of PCs delayed the hydration process. For the XRD patterns of the hydration products after being hydrated for 7 days, the differences in XRD patterns could hardly be distinguished; thus implying that the retardation effect of hydration in the case of the crosslinked-PC was mainly reflected during the early stages. Compared to the hydration products hydrated for 1 day, due to the conversion from the AFt phase to the AF-mono (AFm) phase, the diffraction peaks corresponding to the AFt phase were decreased, and the diffraction signals of Ca(OH)_2_ became strong after hydration [35,36]. The compressive strengths versus different PC dosages are shown in Figure 10b. After hydrating at room temperature for 7 days, blank cement showed a compressive strength of 37.0 MPa. With the addition of PCs, the cement paste exhibited higher dispersive capacity than blank, and a more dense structure of the hydration products was formed. Thus, the cement/PC samples show higher compressive strengths than those of blank cement. Moreover, the compressive strengths of cement/comb-like-PC and cement/crosslinked-PC were similar and the highest compressive strength of 61 and 60 MPa were obtained at the PC dosage of 0.2% bwoc, respectively. When the dosage of PC was over 0.2% bwoc, the compressive strengths of the cement/PC samples decreased with the PC dosage. Compared to comb-like-PC, the crosslinked-PC led to a more significant delayed effect on the early hydration process and sightly affected the later compressive strength of the hydration products.

### 3.8. SEM Analysis of the Hydrated Products

**Figure 11:**
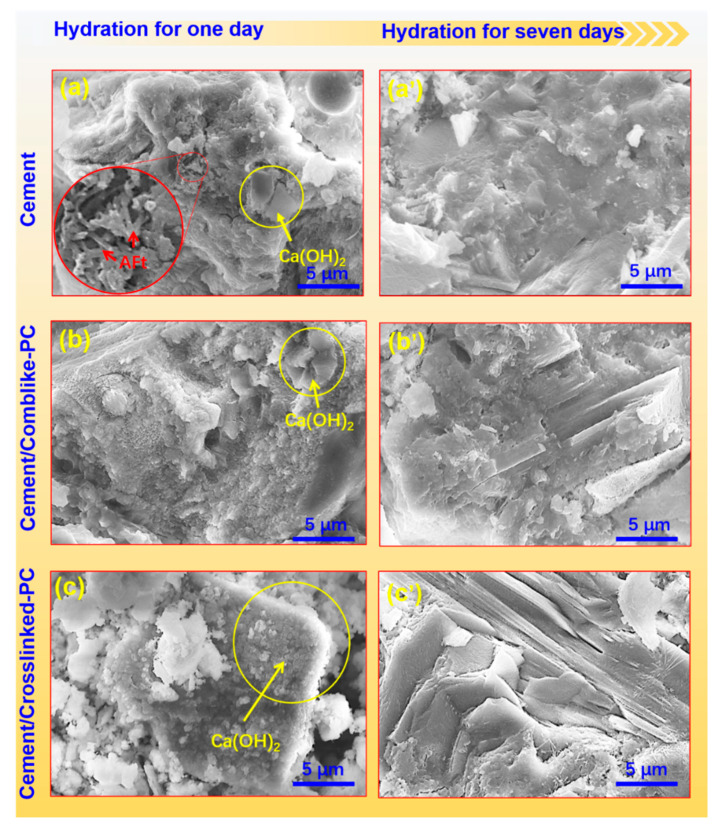
SEM micrographs of cement samples after hydration for 1 day (**a**–**c**) and 7 days (**a’**–**c’**) with a magnification of 5000.

Figure 11a–c show the morphology of the hydrated cement products after 1 day. As observed from Figure 11, both C-S-H and Ca(OH)_2_ crystal were obvious in the cement hydration products. On incorporating the crosslinked-PC in the cement, a large extent of the C-S-H gel was attached on the surface of the Ca(OH)_2_ crystals. During the early hydration stage, the hydration products were needle-like AFt phase, square Ca(OH)_2_ crystal and amorphous C-S-H gel. The cement slurry was in a plastic state, and the porosity was largely retained. However, the crystals were too small to connect with each other to result in a steady state. Comparing the effect of the comb-like-PC and crosslinked-PC on the cement hydration process, it could be concluded that the crosslinked-PC retarded the hydration of cement and provided a dense structure during the hydration process. After hydration for 7 days (Figure 11a’–c’), the hydration products connected with each other to form a less porous network structure, with the paste gaining strength. The SEM analysis further confirmed that the crosslinked-PC was more efficient in retarding the hydration of the cement paste, which was consistent with the WAXD results. It is speculated that a certain amount of the crosslinked-PC can postpone the cement hydration process.

## 4. Conclusions

In this study, a pentaerythritol tetraacrylate-based crosslinked-PC has been successful synthesized. In comparison with the comb-like-PC, the crosslinked-PC exhibited a higher dispersive capacity. At the dosage of 0.6% bwoc, the fluidity of the cement/crosslinked-PC paste was about 340 mm, which was 40~50 mm larger than the cement/comb-like-PC paste. The dispersion retention capacity of the cement/crosslinked-PC paste was observed to be much superior due to higher adsorbed amounts on the cement particles. Moreover, the cement/crosslinked-PC paste exhibited the initial and final setting durations of 196 and 356 min, respectively, which indicated an enhancement of 18 and 68 min compared to the cement/comb-like paste. The crosslinked copolymers exhibit a stronger retardation effect than the comb-like copolymers due to their enhanced adsorbed amounts and stronger steric hindrance effect. Moreover, the compressive strengths of the cement/crosslinked-PC were similar and the highest compressive strength of 60 MPa was obtained at the dosage of 0.2% bwoc. Thus, it is feasible to improve the dispersive capacity and the dispersion retention capacity of PC by changing the molecular structure from comb-like to slightly crosslinked. The findings reported in this study exhibit the potential for expanding the preparation method of the PCs.

## Figures and Tables

**Table 1:** Monomer combination in synthesis of the PCs.

Sample	HPEG (mmol)	KPS (mmol)	AA (mmol)	SAMS (mmol)	PETA (mmol)	TGA (mL)	H_2_O (mL)
Comb-like-PC	20	15	30	10	0	0.5	60
Crosslinked-PC	20	15	30	10	3.2	0.5	60

**Table 2:** The molecular weight and distribution of PCs.

Sample	M_n_	M_w_	PD
Comb-like-PC	27,348	69,706	2.55
Crosslinked-PC	38,882	109,588	2.82

**Table 3:** The cement hydration parameters extracted from the heat evolution curves.

Sample	*t*_A_ (h)	*k*_A–B_	(d_Q_/d_t_)_A_ (mW/g)	(d_Q_/d_t_)_C_ (mW/g)
Cement	2.79	0.25	0.11	0.32
Cement/Comb-like-PC	3.85	0.15	0.07	0.30
Cement/Crosslinked-PC	4.05	0.14	0.05	0.29

## References

[1] Lee T., Lee J., Jeong J., Jeong J. (2021). Improving Marine Concrete Performance Based on Multiple Criteria Using Early Portland Cement and Chemical Superplasticizer Admixture. Materials.

[2] Zhao H., Liao B., Nian F., Zhao Y., Wang K., Pang H. (2018). Synthesis and characterization of a PAMAM dendrimer-based superplasticizer and its effect on the properties in cementitious system. J. Appl. Polym. Sci..

[3] Jimma B.E., Rangaraju P.R. (2015). Chemical admixtures dose optimization in pervious concrete paste selection–A statistical approach. Constr. Build. Mater..

[4] Haruna S., Fall M. (2019). Time- and temperature-dependent rheological properties of cemented paste backfill that contains superplasticizer. Powder Technol..

[5] Ma B., Peng Y., Tan H., Lv Z., Deng X. (2018). Effect of Polyacrylic Acid on Rheology of Cement Paste Plasticized by Polycarboxylate Superplasticizer. Materials.

[6] Zhang Y., Kong M., Lu B., Lu Z., Hou S. (2015). Effects of the charge characteristics of polycarboxylate superplasticizers on the adsorption and the retardation in cement pastes. Cem. Concr. Res..

[7] Li H., Yao Y., Wang Z., Cui S., Wang Y. (2020). Influence of Monomer Ratios on Molecular Weight Properties and Dispersing Effectiveness in Polycarboxylate Superplasticizers. Materials.

[8] Carabba L., Manzi S., Bignozzi M.C. (2016). Superplasticizer Addition to Carbon Fly Ash Geopolymers Activated at Room Temperature. Materials.

[9] Ilg M., Plank J. (2019). Synthesis and Properties of a Polycarboxylate Superplasticizer with a Jellyfish-Like Structure Comprising Hyperbranched Polyglycerols. Ind. Eng. Chem. Res..

[10] Yu P., Pang H., Huang J., Meng Y., Huang H., Li S., Liao B., Liang L. (2020). Synthesis and rapid cement hardening evaluation of polycarboxylate superplasticizers incorporating ester groups in their backbone chain. J. Appl. Polym. Sci..

[11] Zhang J., Wang X., Jin B., Zhang X., Li Z., Guan X. (2021). Effect of superplasticizers on hydration kinetics of ultrafine sulfoaluminate cement-based grouting material. Thermochim. Acta.

[12] Qian Y., De Schutter G. (2018). Different Effects of NSF and PCE Superplasticizer on Adsorption, Dynamic Yield Stress and Thixotropy of Cement Pastes. Materials.

[13] Gelardi G., Sanson N., Nagy G., Flatt R. (2017). Characterization of comb-shaped copolymers by Multidetection SEC, DLS and SANS. Polymers.

[14] Weckwerth S.A., Radke W., Flatt R.J. (2021). A Method for Characterizing the Chemical Heterogeneity of Comb-Copolymers and Its Dependence on Synthesis Routes. Polymers.

[15] Etsuo S., Kazuo Y., Akira O. (2003). Molecular Structure and Dispersion-Adsorption Mechanisms of Comb-Type Superplasticizers Used in Japan, J. Adv. Concr. Technol..

[16] Cai Y., Yang T., Ren Q., Liang M., Zhou S. (2019). Enhanced slump retention using polycarboxylate superplasticizers obtained by micro-crosslinking reaction. Mater. Express.

[17] Liu H., Pang H., Ou J., Zhang L., Dai Y., Liao B. (2014). Effect of cross-linked polycarboxylate-type superplasticizers on the properties in cementitious system. J. Appl. Polym. Sci..

[18] Liu X., Guan J., Lai G., Wang Z., Zhu J., Cui S., Lan M., Li H. (2017). Performances and working mechanism of a novel polycarboxylate superplasticizer synthesized through changing molecular topological structure. J. Colloid Interface Sci..

[19] Lin X., Pang H., Wei D., Lu M., Liao B. (2021). Effect of the cross-linker structure of cross-linked polycarboxylate superplasticizers on the behavior of cementitious mixtures. Colloids Surf. A Physicochem. Eng. Asp..

[20] (2012). Method for Testing Uniformity of Concrete Admixture.

[21] (2002). Standard for Test Method of Performance on Ordinary Fresh Concrete.

[22] (2011). Test Methods for Water Requirement of Normal Consistency, Setting Time and Soundness of the Portland Cement.

[23] Shameli K., Bin Ahmad M., Jazayeri S.D., Sedaghat S., Shabanzadeh P., Jahangirian H., Mahdavi M., Abdollahi Y. (2012). Synthesis and Characterization of Polyethylene Glycol Mediated Silver Nanoparticles by the Green Method. Int. J. Mol. Sci..

[24] Zhao H., Liao B., Nian F., Wang K., Guo Y., Pang H. (2018). Investigation of the clay sensitivity and cement hydration process of modified HPEG-type polycarboxylate superplasticizers. J. Appl. Polym. Sci..

[25] Schennach R., Cocke D., Mollah M., Adams W. (2000). A Review of Cement-Superplasticizer Interactions and Their Models. Adv. Cem. Res..

[26] Plank J., Hirsch C. (2007). Impact of zeta potential of early cement hydration phases on superplasticizer adsorption. Cem. Concr. Res..

[27] Pourchet S., Liautaud S., Rinaldi D., Pochard I. (2012). Effect of the repartition of the PEG side chains on the adsorption and dispersion behaviors of PCP in presence of sulfate. Cem. Concr. Res..

[28] Aggarwal S.L. (1976). Structure and properties of block polymers and multiphase polymer systems: An overview of present status and future potential. Polymer.

[29] Verdonck B., Gohy J.F., Khousakoun E., Jérôme R., Du Prez F. (2005). Association behavior of thermo-responsive block copolymers based on poly(vinyl ethers). Polymer.

[30] Plank J., Yu B. (2010). Preparation of hydrocalumite-based nanocomposites using polycarboxylate comb polymers possessing high grafting density as interlayer spacers. Appl. Clay Sci..

[31] Liu X., Wang Z., Zhu J., Zheng Y., Cui S., Lan M., Li H. (2014). Synthesis, characterization and performance of a polycarboxylate superplasticizer with amide structure. Colloids Surf. A Physicochem. Eng. Asp..

[32] Pourchet S., Comparet C., Nicoleau L., Nonat A. Influence of PC Superplasticizers on Tricalcium Silicate Hydration. Proceedings of the 12th International Congress on the Chemistry of Cement.

[33] Cartuxo F., Brito J., Evangelista L., Jimenez J.R., Ledesma E.F. (2015). Rheological behaviour of concrete made with fine recycled concrete aggregates—Influence of the superplasticizer. Constr. Build. Mater..

[34] Bullard J.W., Jennings H.M., Livingston R.A., Nonat A., Scherer G.W., Schweitzer J.S., Scrivener K.L., Thomas J.J. (2011). Mechanisms of cement hydration. Cem. Concr. Res..

[35] Zhang Y., Kong X. (2014). Influences of superplasticizer, polymer latexes and asphalt emulsions on the pore structure and impermeability of hardened cementitious materials. Constr. Build. Mater..

[36] Pratt P.L., Ghosh A., Skalny J., Hewlett P.C., Hirsch P.B., Birchall J.D., Double D.D., Kelly A., Moir G.K., Pomeroy C.D. (1983). Electron microscope studies of Portland cement microstructures during setting and hardening. Philos. Trans. R. Soc. Lond. Ser. A Math. Phys. Sci..

